# Worldwide evaluation of Clinical Practice Strategies (CliPS) for lung involvement in Still’s disease within the JIR-CliPS network: a COST action

**DOI:** 10.1093/rap/rkaf073

**Published:** 2025-07-03

**Authors:** Maurine Jouret, Francisca Aguiar, Charlotte Girard-Guyonvarc’h, Yulia Vyzhga, Filipa Oliveira-Ramos, Cristina Costa Lana, Rahma Guedri, Alain Lefevre-Utile, Djohra Hadef, Juan Manuel Mosquera Angarita, Seza Ozen, Sezgin Sahin, Soad Hashad, Karima Daghor-Abbaci, Dirk Foell, Sophie Georgin-Lavialle, Katerina Theodoropoulou, JIR-CLIPS Network, Konstantinos Pateras, Jovana Paunovic Pantic, Alexandre Belot, Sezgin Sahin, Sara Ganhão, Joan Calzada-Hernández, Lovro Lamot, Ida Dzifa Dey, Eve Smith, Sylvia Kamphuis, Cristina Lanna, Raffaella Carlomagno, Marleen Verkaaik, Chris Pruunsild, Jade Cognard, Amani Mubarak, Natasha Jordan, Belde Kasap Demir, Angela Migowa, Stephen Marks, Seher Sener, Edoardo Marrani, Phil Riley, Selcan Demir, Valentina Natoli, Giedre Januskeviciute, Patricia Costa Reis, Arune Ramanauskiene, Ausra Snipaitiene, Daiva Gorczyca, Jaanika Ilisson, Jacek Postepski, Judith Sánchez-Manubens, Jurgita Marciulynaite, Mejbri Manel, Teresa Giani, Tilmann Kallinich, Yosef Uziel, Zohra Fitouri, Seza Ozen, Marija Jelusic, Margarita Ganeva, Caroline Platt, Vladimir Iacomi, Zeynep Balik, Emil Aliyev, Nastasia Kifer, Dominik Mueller, Andzelika Slegeryte, Helmut Wittkowski, Inès ELHANI, Juergen Brunner, Marion Delplanque, Meri Kirijas, Roberta Caorsi, Sonia Carriquí Arenas, Tanja Hinze, Véronique Hentgen, Rim Bourguiba, Mario Sestan, Tamás Constantin, Helen Lachmann, Ghalia Khellaf, Yonatan Butbul, Nuray Aktay Ayaz, Stephanie Ducharme-Benard, Fatih Haşlak, Mr Stefan Backes, Muserref Kasap Cuceoglu, Naiera Assalia, Vafa Guliyeva, Anna Mamutova, Amira Cerimagic, Betul Sozeri, Ezgi Deniz Batu, Michaël Hofer, Ciprian Jurcut, Caroline Vinit, Estíbaliz Iglesias, Mariana Rodrigues, Nadezhda Shapovalova, Natasa Toplak, Marco Gattorno, Rawane Dagher, Ricardo Craveiro Costa, Margarida Faria, Katerina Laskari, Gulcan Ozomay Baykal, Hafize Emine Sönmez, Amila Hadzimuratovic, Gvantsa Jajanidze, Sophie Georgin-Lavialle, RAHMA Guedri, Maurine Jouret, Katerina Theodoropoulou, Juan Manuel Mosquera Angarita, Francisca Aguiar, Dirk Foell, Soad Hashad, Djohra Hadef, Alain Lefevre-Utile, Charlotte Girard, Filipa Oliveira Ramos, Karima Daghor-Abbaci, Raquel Faria, Sinisa Savic, Joanne May, Souhaibou NDONGO, Elissar Dagher, Sofia Ferreira Azevedo, Eva Timonet, Philippe Mertz, Wafa Hamdi, Roberta Naddei, Vinay Shivamurthy, Syrine Bellakhal

**Affiliations:** Pediatric Nephrology, Rheumatology, Dermatology Unit, Hospices Civils de Lyon, Lyon, France; Adult and Pediatric Rheumatology Unit, ULS São JoãoYoung, Porto, Portugal; Faculdade de Medicina da Universidade do Porto, Porto, Portugal; Division of Rheumatology, Department of Medicine, University Hospital of Geneva, Geneva, Switzerland & Swiss RITA; National Pirogov Memorial Medical University, Vinnytsya, Ukraine; Division of Rheumatology, The Hospital for Sick Children “SickKids”, Toronto, Canada; Paediatric Rheumatology Unit, University Hospital ULS Santa Maria, Lisbon School of Medicine, University of Lisbon, Lisbon, Portugal; Rheumatology, Medical School of Medicine, Federal University of Minas Gerais State (UFMG), Belo Horizonte, Brazil; Faculty of Medicine of Tunis, Peadiatric Rheumatology Unit, Béchir Hamza Children’s Hospital, Tunis, Tunisia; University of Tunis El Manar, Tunis, Tunisia; Département Femme-Mère-Enfant (DFME), Service de Pédiatrie, Lausanne, Switzerland; Faculté de Biologie et de Médecine de l’Université de Lausanne, Lausanne, Switzerland; Faculty of Medicine, Batna 2 University, Batna, Algeria; Pediatric Rheumatology Unit, Sant Joan de Déu Children’s Hospital, Barcelona, Spain; Department of Pediatrics, Hacettepe University, Ankara, Turkey; Department of Pediatric Rheumatology, University Cerrahpasa, Istanbul, Turkey; Department of Pediatric Rheumatology, Tripoli Children Hospital, University of Tripoli, Tripoli, Libya; Department of Internal Medicine, Algeria Rheumatology and Immunology, Bab El Oued University Hospital Center, University of Algiers, Algiers, Algeria; Pediatric Rheumatology and Immunology, University Children’s Hospital Münster, Münster, Germany; Internal Medicine Department, Sorbonne University, Tenon Hospital, AP-HP, DMU3ID, Paris, France; CEREMAIA & ERN RITA, Paris, France; Unit of Pediatric Immunology, Allergology and Rheumatology, Lausanne University Hospital (CHUV) and University of Lausanne (UNIL), Lausanne, Switzerland

Dear Editor, Still’s disease is a rare autoinflammatory condition encompassing systemic juvenile idiopathic arthritis (sJIA) and adult-onset Still’s disease (AOSD). Still’s disease can be complicated by lung inflammation (LD), which appears to be more prevalent than previously recognized. Reports indicate that 6.8% of children with sJIA develop LD within 2 years of onset, with an initial mortality rate as high as 68% [1–3]. In adults, 5–12% of Still’s disease patients develop LD, with similarly high mortality rates [4, 5]. Recent findings from the AIDA Network registry published in your journal have provided new insights into the extent and characteristics of LD-Still’s disease [6]. In this cohort of 90 adult and pediatric patients, parenchymal lung involvement was observed in 34.4% and pulmonary arterial hypertension in 2.3% [6]. The latest EULAR/PReS guidelines have provided specific recommendations for the management of LD in Still’s disease [7]. While progress has been made in understanding these complications, significant gaps persist in clinical practice worldwide.

To evaluate global clinical approaches to LD in Still’s disease, the Juvenile Inflammatory Rheumatism Network conducted an international survey under Clinical Practice Strategy (CliPS) initiative. This survey has been distributed since September 2022 and targeted clinicians from diverse healthcare settings and experience levels (Supplementary Table S1, available at *Rheumatology Advances in Practice* online), collecting data on demographic characteristics, diagnostic methodologies, and therapeutic strategies. The responses were analyzed in September 2024, before the publication of EULAR/PReS recommendations, and stratified by pediatric and adult specialists, considering that clinical practices remain heterogeneous between these groups.

Among 372 clinicians from five continents (Supplementary Fig. S1, available at *Rheumatology Advances in Practice* online), who participated in the survey, 69 respondents (18.5%)—57 paediatricians and 12 adult specialists—reported experience in managing LD in Still’s disease. The survey results are detailed in Table 1.

**Table 1.** Diagnostic investigations from paediatricians for lung involvement in Still’s disease

Investigation/action	Always (%)	Sometimes (%)	Never (%)	Number of participants (*n*)	Non respondent
Chest radiography	94	6	0	50/57 (88%)	*n* = 7/57
Chest CT scan	79	21	0	53/57 (93%)	*n* = 4/57
Echocardiography (for pulmonary hypertension)	82	18	0	50/57 (88%)	*n* = 7/57
Bronchoalveolar lavage (BAL)	24	63	13	46/57 (80%)	*n* = 11/57
Lung biopsies	2	72	26	43/57 (75%)	*n* = 10/57
Cytokine assays	19.2	53.8	26.9	41/57 (72%)	*n* = 16/57
IFN signature	15	42	43	40/57 (70%)	*n* = 17/57
Whole-exome sequencing	5	62	33	40/57 (70%)	*n* = 17/57
IL-18 measurements (for follow-up in LD-Still’s Disease)	Yes: 28	–	No: 71		*n* = 32/32 (100%)
HLADRB1*15 as a supplementary argument for lung involvement	40	16	44		*n* = 32/32 (100%)
Change in therapeutic approach if HLADRB1*15 allele is identified	29	23	48		*n* = 31/32 (96,8%)

A total of 79% and 82% of paediatricians reported routinely using chest CT scans and echocardiography for pulmonary assessment, respectively. The use of biomarkers such as IL-18 is not yet standard practice, with only 28% of paediatricians reporting its use. The interferon signature, while not yet universally adopted, is gaining recognition, with 57% of paediatricians incorporating it into clinical practice.

Genetic testing for HLA-DRB1*15, a potential risk factor for drug-induced reactions [1], is inconsistently performed, with 56% of paediatricians utilizing it, showing significant regional variation (Supplementary Table S2, available at *Rheumatology Advances in Practice* online). However, the clinical significance of HLA typing remains controversial in the literature [3, 7] and raises concerns among most practitioners.

Regarding treatment, most paediatricians (*n* = 38/46, 83%) opted not to discontinue IL-1 or IL-6 inhibitors if LD developed. Of these, 56% (*n* = 26/46) reported switching biologics if the treatment was ineffective in controlling disease activity, while the remaining continued the same biologic with the addition of other therapeutics. A significant proportion of paediatricians have integrated JAK inhibitors into their clinical practice for the treatment of LD, with 38.8% of participants selecting this option, followed by T cell-targeted therapies such as ciclosporin or mycophenolate mofetil (Supplementary Figs S2 and S3, available at *Rheumatology Advances in Practice* online). Hematopoietic stem cell transplantation remains a last-resort option, with 82% of surveyed paediatricians considering it a viable approach for refractory cases.

The survey results, which focused on a pediatric point of view due to the low participation of adult specialists, revealed substantial global variation. The asymptomatic development of these conditions often complicates early detection [8]. Chest CT scans, radiography and echocardiography appear to be standard tools for early detection, aligning with EULAR/PReS recommendations. Pulmonary biopsies, though informative, seem to be infrequently performed due to their invasive nature. We suggest that this procedure could be reserved for complex cases.

A notable finding is the disparity in the use of IL-18, which is less frequently performed in emerging countries, likely due to cost and accessibility constraints. The interferon signature, although not officially recognized as a biomarker in EULAR/PReS guidelines [7] or registry data [6], appears to be increasingly used in clinical practice, particularly with the growing adoption of JAK inhibitors, which directly target interferon signalling pathways.

Despite concerns that biologics might have exacerbated LD [8], most paediatricians continued their use in alignment with EULAR/PReS guidelines. Notably, data from the AIDA registry found no significant association between IL-1 or IL-6 inhibitors and the development of LD [6]. Our study also highlights the increasing incorporation of T cell-targeted therapies in cases of LD-associated Still’s disease, suggesting a growing emphasis on combination therapy as recommended by the EULAR/PreS.

Moreover, adult specialists were underrepresented in our study, suggesting they may be less familiar with LD in Still’s disease compared with paediatric rheumatologists. This result is opposed to the AIDA registry data collecting mostly adult patients (86.7%).

In conclusion, this study highlights gaps between registry data, recent guidelines and real-world clinical practices in managing LD associated with Still’s disease. Bridging these gaps will require targeted strategies to enhance clinician awareness, improve access to diagnostic tools and validate treatment recommendations across diverse healthcare settings.

## Supplementary Material

rkaf073_Supplementary_Data

## Data Availability

The data underlying this article cannot be shared publicly due to the privacy protection of individuals that participated in the study. The data will be shared on reasonable request to the corresponding author.
