# Supplementary material for: Worldwide evaluation of Clinical Practice Strategies (CliPS) for lung involvement in Still’s disease within the JIR-CliPS network: a COST action
Source: Rheumatol Adv Pract. 2025 Jul 3;9(3):rkaf073. doi: 10.1093/rap/rkaf073 (PMC12401577; doi:10.1093/rap/rkaf073)
Supplement: rkaf073_Supplementary_Data [file rkaf073_supplementary_data.docx]

Supplementary material

Supplementary Table S1 : Demographic data from the survey on clinical practices of pediatricians in Still’s Disease. *N* represents the number of participants.

| Results of the survey about Clinical Practices of pediatricians in Still’s Disease | N= Number of participants |
| --- | --- |
| Demographics of participants following at least one patient with LD  Sex ratio  Specialties (n=69)  Paediatrician  Rheumatologist  Pediatric and adult rheumatologist  Internal medicine  Type of patients cared for (n=69)  Only children  Both adult and children  Adult    Years of experience of pediatricians (n=57)  More than 10 years  Between 5 and 9 years  Less than 4 years  Type of institution of pediatricians (n=57)  Tertiary or university Hospital  Hospital  Private practice | 0.7M/1F    57 (76%)  6 (10%)  5 (9%)  3 (5%)  48 (70%)  12 (17%)  9 (13%)  32 (56%)  17 (30%)  8 (14%)  49 (84%)  8 (14%)  1 (2%) |


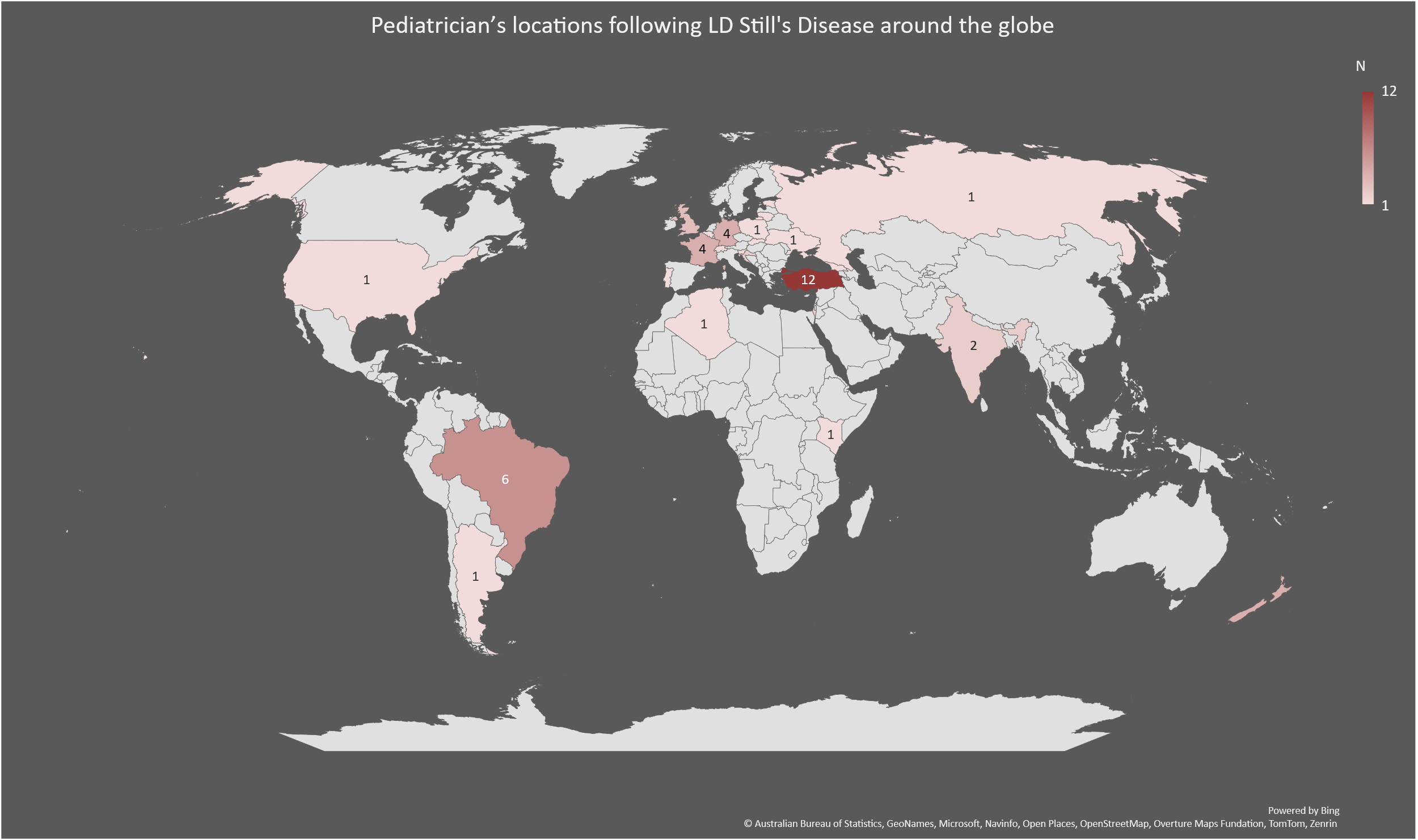


Supplementary Figure S1: Paediatrician’s locations around the globe. Only participants taking care of patients with LD-Still’s Disease were represented.

Alternative text: This figure presents the geographic distribution of participants. Europe had a strong presence, covering both Eastern and Western regions, with notable contributions from Turkey (n=12), Germany (n=4), and France (n=4). Two participants were from Africa, while South America was well represented, with seven physicians from Brazil (n=6) and Argentina (n=1). Additionally, four participants were from New Zealand. In contrast, the United States had minimal representation, with only one participant, likely due to the limited reach of the JIR network in North America.


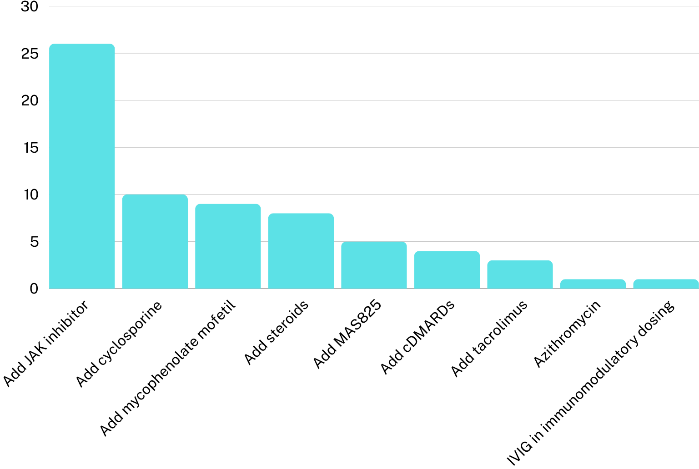

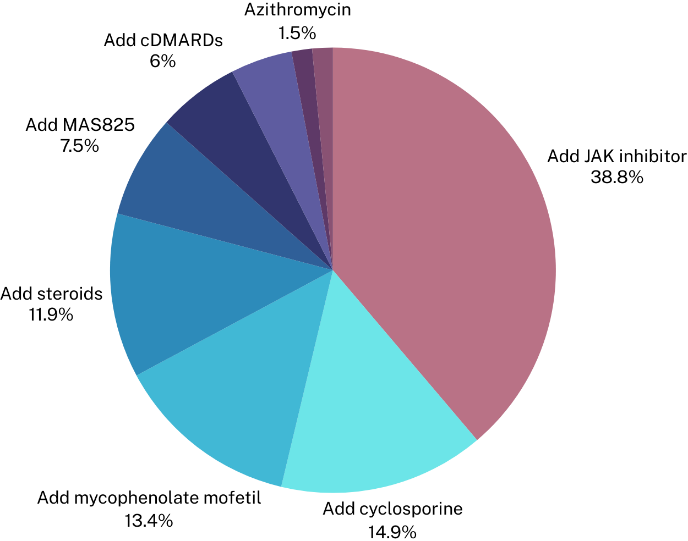


Supplementary Figure S2 and S3: Frequency of treatments selected by pediatricians (n=31) for managing lung involvement in Still’s disease (multiple selections allowed).

Alternative text: This figure illustrates pediatricians' treatment preferences for lung involvement in Still’s disease. JAK inhibitors are the most commonly chosen treatment, followed by cyclosporine and mycophenolate mofetil.

Supplementary Table S2: Location of pediatricians regarding management of lung involvement in Still’s disease.

| Clinical practice | Yes and Sometimes | No |
| --- | --- | --- |
| IL-18 measurement  n=32 | France n=3  Italy n=2  Belgium n=1  Germany n=1  Portugal n=1  United States n=1  Total n=9 | Turkey n=6  Brazil n=2  Germany n=2  Algeria n=1  Poland n=1  Switzerland n=1  Slovenia n=1  Slovakia n=1  Russian Federation n=1  Lithuania n=1  New Zealand n=1  Argentina n=1  Kenya n=1  India n=1  Georgia n=1  Estonia n=1  United Kingdom n=1  Total n=23 |
| HLADRB1*15  n=32 | Turkey n=6  France n=4  Argentina n=1  Belgium n=1  Germany n=1  Italy n=1  Portugal n=1  Slovakia n=1  Slovenia n=1  United Kingdom n=1  Total n=18 | Brazil n=2  Germany n=1  Algeria n=1  Estonia n=1  Georgia n=1  India n=1  Israel n=1  Italy n=1  Lithuania n=1  New Zealand n=1  Poland n=1  Russian Federation n=1  Ukraine n=1  Total n=14 |

**Always/Sometimes/Never:** Percentages represent how often pediatricians report performing these investigations or actions. **Number of Participants (n):** Refers to the number of pediatricians who answered the specific question in the survey.
